# JGR-NMF: joint graph-regularized non-negative matrix factorization for spatial domain identification

**DOI:** 10.7717/peerj.20585

**Published:** 2026-02-10

**Authors:** Juan Liang, Jiuxi Huang, Chenxi Xi, Yun Wang, Juntao Li

**Affiliations:** 1 School of Computer Science and Technology, Henan Institute of Technology, Xinxiang, Henan, China; 2 School of Mathematics and Statistics, Henan Normal University, Xinxiang, Henan, China

**Keywords:** Spatial transcriptomics, Non-negative matrix factorization, Adjacency matrix

## Abstract

The spatial transcriptomics technique provides an unprecedented perspective for analyzing the distribution patterns of cells within tissues and their functional tissue structures. To enhance the accuracy and robustness of spatial domain identification, we propose Joint Graph-Regularized Non-negative Matrix Factorization (JGR-NMF). An adaptive neighborhood graph construction strategy is introduced by applying an *n*th-power transformation to the spot adjacency probability matrix, thereby automatically optimizing the neighborhood size for individual spots. Furthermore, a JGR-NMF framework is developed, integrating this adaptively constructed kNN graph with the spatial adjacency matrix. Evaluations conducted on two breast cancer datasets, one Mouse Kidney dataset and one Mouse Embryo dataset, demonstrate that JGR-NMF significantly outperforms five state-of-the-art baseline methods in spatial domain identification. Systematic ablation studies further confirm the critical role of graph regularization in enhancing model performance.

## Introduction

Spatial transcriptomics has become a transformative technique for analyzing gene expression in a spatial context (Anderson et al., 2022; Jin et al., 2024; Liang et al., 2024a). The relevant experimental platforms can be broadly categorized into sequencing-based approaches (*e.g.*, 10x Visium (Ji et al., 2020) and imaging-based methods (*e.g.*, seqFISH (Lubeck et al., 2014), MERFISH (Chen et al., 2015)), with the latter often achieving single-cell resolution. Although these techniques differ in terms of resolution and throughput, they both generate high-dimensional spatial datasets, which are crucial for analyzing tissue structures. A fundamental goal in the analysis of spatial transcriptomic data is the identification of spatial domains, in which tissue sections are partitioned into functionally distinct regions through the integration of gene expression patterns and their spatial context. Accurate identification of these spatial domains not only reveals heterogeneity within tissue microenvironments but also facilitates the investigation of spatially dependent molecular features associated with disease mechanisms, such as the progressive remodeling of tumor microenvironments (Wang et al., 2024c). However, inherent challenges posed by high-dimensional gene expression data and complex spatial dependencies hinder effective fusion of these modalities, thereby impeding robust classification of spatial domains.

In the realm of single-cell RNA sequencing (scRNA-seq) data analysis, K-means clustering (Sinaga & Yang, 2020) and Louvain (Zhang et al., 2021) algorithms have emerged as two widely utilized classical unsupervised clustering techniques. Currently, these algorithms have been successfully integrated into prominent single-cell analysis platforms such as SCANNPY (Wolf, Angerer & Theis, 2018) and Seurat (Stuart et al., 2019), demonstrating commendable performance in addressing cellular heterogeneity. However, when these traditional methods are directly applied to spatial transcriptomics data analysis, they tend to overlook the spatial location information of spots, relying solely on gene expression similarity for clustering. This limitation may result in the misclassification of spatially adjacent spots that exhibit subtle differences in their transcriptomes. Furthermore, the inherent technical noise and sparsity present in spatial transcriptome data further compromise the robustness of these approaches. These limitations have motivated the development of a new generation of spatial domain identification algorithms that can effectively integrate spatial information.

To address the aforementioned technical challenges, a series of innovative clustering algorithms that incorporate spatial information have been developed, significantly enhancing the accuracy of spatial domain identification. The BayesSpace (Zhao et al., 2021) algorithm, which is based on a Bayesian statistical framework, effectively integrates spatial neighborhood structures, enabling high-resolution spatial domain analysis at the subcellular level. The BANKSY (Singhal et al., 2024) method utilizes a weighted spatial graph network to systematically combine single-cell gene expression profiles with neighborhood features, thereby successfully revealing complex cellular spatial patterns and tissue microenvironment structures. The SpaGCN (Hu et al., 2021) algorithm, leveraging graph convolutional networks, demonstrates superior performance in maintaining consistency between gene expression patterns and histological features through spatial neighborhood feature aggregation. Notably, the MNMST (Wang, Liu & Ma, 2024) algorithm employs a multi-layer isomorphic network architecture to achieve joint representation learning of spatial and expression data, while spaMMCL (Liang et al., 2024b) introduces a multimodal learning framework that integrates gene expression, histological images, and spatial context in a unified manner. Despite these advancements, several critical challenges remain. First, although deep learning and Bayesian inference-based methods achieve high accuracy, their computational complexity hinders scalability for large-scale datasets. Second, the inherent “black-box” nature of deep learning models compromises interpretability, making it difficult to elucidate underlying biological mechanisms. Therefore, the development of novel algorithms that can simultaneously ensure computational efficiency, interpretability, and generalizability remains a crucial research direction in the field of spatial transcriptomics data analysis.

Non-negative matrix factorization (NMF) can achieve additive combination of data due to the non negative constraints it imposes, thereby decomposing into factors with interpretability (Takasawa et al., 2024). This characteristic makes NMF particularly suitable for extracting basis vectors with clear biological significance from sparse high-dimensional genomic data, and thus NMF has been widely applied in the cluster analysis of transcriptome data (Shiga et al., 2021). For example, Wang et al. (2024) used the NMF method to cluster the cancer-associated fibroblasts in prostate cancer and identified key subtypes related to antigen presentation and immune regulation. On the basis of classical NMF, Graph Regularized NMF (GNMF) further incorporates the similarity graph structure among samples to promote the geometric proximity of adjacent samples in the latent factor space, thereby enhancing the model’s perception of the intrinsic manifold structure of the data. In addition, GNMF also provides an effective approach for integrating multi-source information (Townes & Engelhardt, 2023). For instance, Li, Xiang & Wei, (2024) proposed the TGR-NMF method to identify disease-associated spatial domains in breast cancer spatial transcriptomics data. This approach combines both gene expression-based neighborhood topology and spatial location-based neighborhood topology to generate a low-dimensional representation of the data, thereby enhancing the accuracy of spatial domain detection. However, the method has been primarily optimized for breast cancer tissues, and its generalizability to other tissue types or disease contexts remains to be fully validated.

To improve the accuracy and robustness of spatial domain identification, we propose a Joint Graph-Regularized Non-negative Matrix Factorization (JGR-NMF) method. Recognizing the critical role of gene expression and spatial location information in spatial domain identification, our approach constructs an adaptive nearest-neighbor graph and a spatial adjacency matrix to precisely capture gene expression patterns and spatial relationships among spots. Specifically, the adaptive nearest-neighbor graph is built using gene expression profiles, where the nth-power transformation of the spot adjacency probability matrix dynamically optimizes the neighborhood size for each spot, eliminating weak connections and thereby enhancing the graph topology. To further enrich spatial feature representation, we integrate spatial location information through a spatial adjacency matrix, which is jointly incorporated with the adaptive nearest-neighbor graph into a unified JGR-NMF framework. Extensive experiments were conducted on two breast cancer datasets, one Mouse Kidney dataset and one Mouse Embryo dataset, benchmarking JGR-NMF against five state-of-the-art methods using the Adjusted Rand Index (ARI), Normalized Mutual Information (NMI), and Purity (PUR) as evaluation metrics. The results demonstrate the superior performance of our method. Rigorous ablation studies further confirm the significant contribution of graph regularization to spatial domain identification accuracy.

## Materials and Methods

### Overview of JGR-NMF

**Figure 1:**
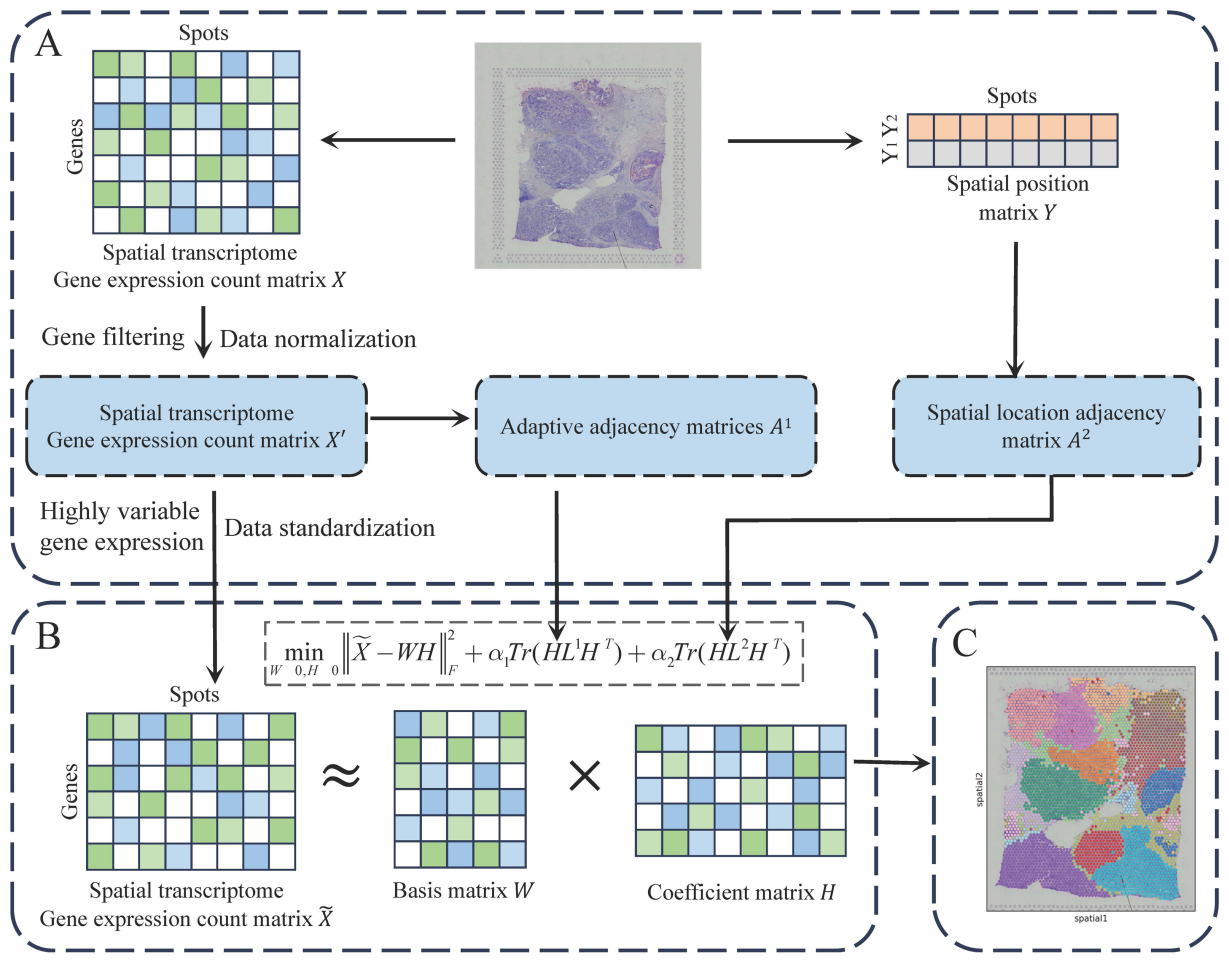
The workflow of JGR-NMF. (A) Obtain the preprocessed gene expression matrix, adjacency matrix *A*^1^, and adjacency matrix *A*^2^ based on the gene expression count matrix and spatial position matrix, respectively. (B) Construct the JGR-NMF model based on the data obtained in (A). (C) Perform K-means clustering on the coefficient matrix decomposed from the JGR-NMF model to obtain spatial domain identification results.

The JGR-NMF method integrates gene expression profiles with spatial coordinates to analyze spatial transcriptomics data. As illustrated in Fig. 1, the analysis begins with data preprocessing, where genes with low expression are filtered, read counts are normalized, and highly variable genes are selected. Subsequently, an adaptive gene expression similarity graph and a fixed spatial proximity graph are constructed to model relationships between spots. These graphs are incorporated into a joint non-negative matrix factorization (NMF) framework, enabling the expression matrix to be factorized while preserving both transcriptional similarity and spatial neighborhood structures. Finally, the low-dimensional embeddings are clustered to identify spatially coherent tissue domains. By jointly leveraging molecular and spatial constraints, JGR-NMF demonstrates improved performance in tissue domain discovery.

### Data description

Four spatial transcriptomics datasets were used in this study, including two cancer datasets (Breast Cancer-1 dataset and Breast Cancer-2 dataset), one Mouse Kidney dataset and one Mouse Embryo dataset. The Breast Cancer-1 dataset was obtained from the 10x Visium platform, with ground truth labels based on the 20 regions defined by Xu et al. (2024). The Breast Cancer-2 dataset was also derived from the 10x Visium platform, primarily consisting of ductal carcinoma *in situ* and invasive carcinoma, with its ground truth labels divided into eight categories using the platform-provided GraphClust algorithm. The Mouse Kidney dataset with six ground truth labels was also downloaded from the 10x Visium platform. The Mouse Embryo dataset was provided by Lohoff et al. (2022). Unlike the previous three datasets, the Mouse Embryo data was generated by the seqFISH platform, offering single-cell resolution and containing 23 cell types. The basic information of the four datasets is presented in Table 1.

**Table 1:** Detailed information of the four datasets.

Dataset	Platform	Spots	Domains
Breast cancer-1 dataset	Visium fresh-frozen	3,798	20
Breast cancer-2 dataset	Visium FFPE	2,518	8
Mouse kidney dataset	Visium fresh-frozen	1,438	6
Mouse embryo dataset	seqFISH	10,150	23

### Data preprocessing

To ensure data quality and provide a reliable foundation for downstream analyses, spatial transcriptomics data are preprocessed through four main steps: gene filtering, data normalization, highly variable gene (HVG) selection, and data standardization.

Let *X* = [*X*_1_, *X*_2_, …, *X*_*i*_, …, *X*_*s*_] ∈ ℝ^*m*×*s*^ denote the gene expression count matrix, where *m* is the number of genes and *s* is the number of spatial spots. Each column vector *X*_*i*_ = [*X*_*i*1_, *X*_*i*2_, …, *X*_*im*_]^*T*^ represents the gene expression profile of the *i*-th spot. Gene filtering is first applied to remove low-quality or uninformative genes. A gene is discarded if it is not expressed in more than 80% of the spots. The zero-expression ratio for gene *j* is computed as $ze{x}_{j}= \frac{1}{s} {\mathop{\sum }\nolimits }_{i=1}^{s}\mathbb{I}({X}_{ij}=0)$, where 𝕀(⋅) is the indicator function that returns 1 if *X*_*ij*_ = 0, and 0 otherwise. Genes with *zex*_*j*_ ≥ *τ* (threshold *τ* = 0.8) are filtered out, resulting in a reduced matrix *X*′ ∈ ℝ^*m*′×*s*^.

To eliminate technical variability between spots, the filtered expression matrix is normalized so that the gene expression values in each spot sum to one: ${X}_{ij}^{{^{\prime}}}\leftarrow {X}_{ij}^{{^{\prime}}}/{\mathop{\sum }\nolimits }_{j=1}^{{m}^{{^{\prime}}}}{X}_{ij}^{{^{\prime}}}$. The resulting matrix *X*′ is used for HVG selection. Highly variable genes are selected to reduce dimensionality while preserving biological signal. For each gene *j*, the expression variance is calculated as ${\sigma }_{j}^{2}= \frac{1}{s} {\mathop{\sum }\nolimits }_{i=1}^{s}({X}_{ij}^{{^{\prime}}}-{\mu }_{j})^{2}$, where ${\mu }_{j}= \frac{1}{s} {\mathop{\sum }\nolimits }_{i=1}^{s}{X}_{ij}^{{^{\prime}}}$ is the mean expression of gene *j*. The top $\widetilde {m}$ genes with the highest variances (typically $\widetilde {m}=2000$) are retained, resulting in the HVG matrix $\widetilde {X}\in {\mathbb{R}}^{\widetilde {m}\times s}$.

Finally, the HVG matrix $\widetilde {X}$ is standardized by scaling the expression profile of each gene across spots to the range [0, 1], using the transformation ${\widetilde {X}}_{ij}\leftarrow {\widetilde {X}}_{ij}/\max ({\widetilde {X}}_{j})$, where $\max ({\widetilde {X}}_{j})$ is the maximum expression value of gene *j* across all spots. The resulting standardized matrix $\widetilde {X}$ is ready for downstream analyses.

### Adaptive neighborhood graph based on spot relationships

The gene expression-based neighborhood graph in spatial transcriptomics data helps identify spots with similar gene expression patterns. Traditional neighborhood graphs typically require each data point to connect to a fixed number of nearest neighbors, regardless of whether those neighbors are located at the center or boundary of a cluster. However, for data points near the cluster boundary, their nearest neighbors may belong to unrelated clusters. Inspired by Cai, Huang & Yin (2022) and Li, Shyr & Liu (2024), an adaptive neighborhood graph is constructed that automatically adjusts the number of neighbors for each spot.

To characterize the positional relationships between spots, the gene expression distance between spots *i* and *i*′ is defined as ${d}_{i{i}^{{^{\prime}}}}^{1}=\parallel {{X}^{{^{\prime}}}}_{i}-{{X}^{{^{\prime}}}}_{{i}^{{^{\prime}}}}\parallel =\sqrt{{\mathop{\sum }\nolimits }_{j=1}^{m}({{X}^{{^{\prime}}}}_{ij}-{{X}^{{^{\prime}}}}_{{i}^{{^{\prime}}}j})^{2}}$. Based on this expression distance, the neighbor set of spot *i* can be derived.

Let *E* = (*E*_*ii*′_)_*s*×*s*_ = [*E*_1_, *E*_2_, …, *E*_*i*_, …, *E*_*s*_] denote the probability matrix representing neighbor connections between spots, where *E*_*i*_ = [*E*_*i*1_, …, *E*_*ii*′_, …, *E*_*is*_]^*T*^ is the connection probability vector from spot *i* to all other spots. The value *E*_*ii*′_ represents the probability that spot *i* connects to spot *i*′, and is inversely proportional to ${d}_{i{i}^{{^{\prime}}}}^{1}$—the smaller ${d}_{i{i}^{{^{\prime}}}}^{1}$, the larger *E*_*ii*′_.

**Figure 2:**
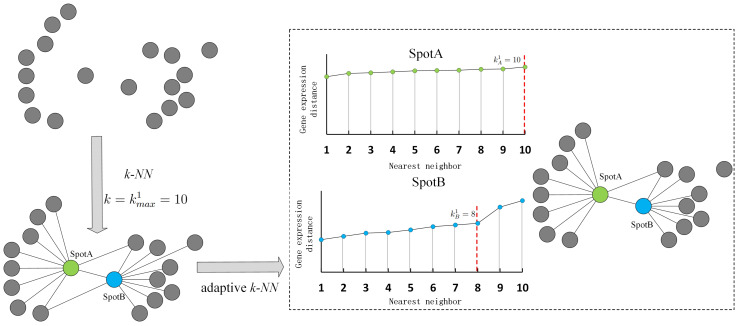
Illustration of adaptive *k*-nearest neighbors.

A maximum number of nearest neighbors ${k}_{max}^{1}$ is set for all spots to compute the distance distribution within the maximum neighborhood and adjust the actual number of neighbors according to sudden changes in distance. If the distance to the $({k}_{i}^{1}+1)$-th neighbor is significantly larger than that to the ${k}_{i}^{1}$-th neighbor, then the true number of neighbors for spot *i* is ${k}_{i}^{1}$ (${k}_{i}^{1}< {k}_{max}^{1}$). Figure 2 shows an example of adaptive *k*-nearest neighbors. In practice, the connection probability is employed instead of distance to identify the true number of nearest neighbors.

Following Cai, Huang & Yin (2022), the probabilities of spots connecting to their neighbors are computed by minimizing an objective function defined as follows.

(1) \begin{eqnarray*}\min _{{E}_{i{i}^{{^{\prime}}}}} & \sum _{i=1}^{s}\sum _{{i}^{{^{\prime}}}=1}^{s}\sum _{n=0}^{\infty } \left( {d}_{i{i}^{{^{\prime}}}}^{1}{E}_{i{i}^{{^{\prime}}}}^{n} \right) & \mathrm{s.t.}  & 0\leq {E}_{i{i}^{{^{\prime}}}}\leq 1, & \sum _{{i}^{{^{\prime}}}=1}^{s}{E}_{i{i}^{{^{\prime}}}}=1, i=1,2,\ldots ,s.\end{eqnarray*}

Since ${\mathop{\sum }\nolimits }_{n=0}^{\infty }{E}_{i{i}^{{^{\prime}}}}^{n}={\lim }_{n\rightarrow \infty } \frac{1-{E}_{i{i}^{{^{\prime}}}}^{n}}{1-{E}_{i{i}^{{^{\prime}}}}} \approx \frac{1}{1-{E}_{i{i}^{{^{\prime}}}}} $ (Wu et al., 2019), Eq. (1) simplifies to

(2) \begin{eqnarray*}\begin{array}{@{}ll@{}} \displaystyle \min _{{E}_{i{i}^{{^{\prime}}}}}&\displaystyle \sum _{i=1}^{s}\sum _{{i}^{{^{\prime}}}=1}^{s} \frac{{d}_{i{i}^{{^{\prime}}}}^{1}}{1-{E}_{i{i}^{{^{\prime}}}}} \\ \displaystyle \mathrm{s.t.} &\displaystyle 0\leq {E}_{i{i}^{{^{\prime}}}}\leq 1,\\ \displaystyle &\displaystyle \sum _{{i}^{{^{\prime}}}=1}^{s}{E}_{i{i}^{{^{\prime}}}}=1, i=1,2,\ldots ,s. \end{array}\end{eqnarray*}

The Lagrangian function of the minimization problem (2) is given by

\begin{eqnarray*}L(E,\zeta ,\lambda )=\sum _{i=1}^{s}\sum _{{i}^{{^{\prime}}}=1}^{s} \frac{{d}_{i{i}^{{^{\prime}}}}^{1}}{1-{E}_{i{i}^{{^{\prime}}}}} -\sum _{i=1}^{s}{\zeta }_{i}(\sum _{{i}^{{^{\prime}}}=1}^{s}{E}_{i{i}^{{^{\prime}}}}-1)-\sum _{i=1}^{s}\sum _{{i}^{{^{\prime}}}=1}^{s}{\lambda }_{i{i}^{{^{\prime}}}}{E}_{i{i}^{{^{\prime}}}}, \end{eqnarray*}

where *ζ* and *λ* are Lagrange multipliers. Taking the derivative of *L*(*E*, *ζ*, *λ*) with respect to *E*_*ii*′_ and setting it to zero, we get

\begin{eqnarray*} \frac{\partial L}{\partial {E}_{i{i}^{{^{\prime}}}}} = \frac{{d}_{i{i}^{{^{\prime}}}}^{1}}{(1-{E}_{i{i}^{{^{\prime}}}})^{2}} -{\zeta }_{i}-{\lambda }_{i{i}^{{^{\prime}}}}=0. \end{eqnarray*}

Using the Karush-Kuhn–Tucker (KKT) conditions (Chen, Zeng & Pan, 2022), the optimal solution to the problem is given by ${E}_{i{i}^{{^{\prime}}}}={ \left( 1- \frac{\sqrt{{d}_{i{i}^{{^{\prime}}}}^{1}}}{\sqrt{{\zeta }_{i}}} \right) }_{+}$, where (⋅)_+_ denotes the positive part, *i.e.,* values where *E*_*ii*′_ > 0. According to the imposed constraints, it follows that

\begin{eqnarray*}\sum _{{i}^{{^{\prime}}}=1}^{s}{ \left( 1- \frac{\sqrt{{d}_{i{i}^{{^{\prime}}}}^{1}}}{\sqrt{{\zeta }_{i}}} \right) }_{+}=1, \mathrm{for}i=1,2,\ldots ,s. \end{eqnarray*}

Assume that spot *i* has ${k}_{i}^{1}$ nearest neighbors such that $ \left( 1- \frac{\sqrt{{d}_{i{i}^{{^{\prime}}}}^{1}}}{\sqrt{{\zeta }_{i}}} \right) > 0$, and each spot can have at most ${k}_{\mathrm{max}}^{1}$ neighbors. Let the distances from spot *i* to all other spots be sorted in ascending order as $[{\widehat{d}}_{i1}^{1},\ldots ,{\widehat{d}}_{il}^{1},\ldots ,{\widehat{d}}_{is}^{1}]$. Then, the constraint can be rewritten as ${\mathop{\sum }\nolimits }_{l=1}^{{k}_{i}^{1}} \left( 1- \frac{\sqrt{{\widehat{d}}_{il}^{1}}}{\sqrt{{\zeta }_{i}}} \right) =1.$ Define ${\mathop{\sum }\nolimits }_{l={k}_{i}^{1}+1}^{{k}_{\mathrm{max}}^{1}} \left( 1- \frac{\sqrt{{\widehat{d}}_{il}^{1}}}{\sqrt{{\zeta }_{i}}} \right) =\sigma ,$ where −1 < *σ* ≤ 0. Then we have ${\mathop{\sum }\nolimits }_{l=1}^{{k}_{\mathrm{max}}^{1}} \left( 1- \frac{\sqrt{{\widehat{d}}_{il}^{1}}}{\sqrt{{\zeta }_{i}}} \right) =1+\sigma .$

Solving for $\sqrt{{\zeta }_{i}}$ yields $\sqrt{{\zeta }_{i}}= \frac{{\mathop{\sum }\nolimits }_{l=1}^{{k}_{\mathrm{max}}^{1}}\sqrt{{\widehat{d}}_{il}^{1}}}{{k}_{\mathrm{max}}^{1}-1-\sigma } .$ Substituting this expression into the optimal solution, we obtain

\begin{eqnarray*}{E}_{i{i}^{{^{\prime}}}}= \left\{ \begin{array}{@{}ll@{}} \displaystyle \frac{\sum _{l=1}^{{k}_{\mathrm{max}}^{1}}\sqrt{{\widehat{d}}_{il}^{1}}-({k}_{\mathrm{max}}^{1}-1-\sigma )\sqrt{{d}_{i{i}^{{^{\prime}}}}^{1}}}{\sum _{l=1}^{{k}_{\mathrm{max}}^{1}}{\widehat{d}}_{il}^{1}} , &\displaystyle \mathrm{if}{d}_{i{i}^{{^{\prime}}}}^{1}\leq {\widehat{d}}_{i{k}_{\mathrm{max}}^{1}}^{1},\\ \displaystyle 0, &\displaystyle \mathrm{otherwise}. \end{array} \right. \end{eqnarray*}

Here, ${\widehat{d}}_{i{k}_{max}^{1}}^{1}$ represents the distance between spot *i* and its ${k}_{max}^{1}$-th nearest neighbor. The condition ${d}_{i{i}^{{^{\prime}}}}^{1}\leq {\widehat{d}}_{i{k}_{max}^{1}}^{1}$ ensures that only the top ${k}_{max}^{1}$ neighbors receive nonzero connection probabilities. Since *E*_*ii*′_ > 0, the number of selected neighbors ${k}_{i}^{1}$ for spot *i* can be determined from Li, Shyr & Liu (2024) as

(3) \begin{eqnarray*}{k}_{i}^{1}= \left\{ \begin{array}{@{}ll@{}} \displaystyle {k}_{\mathrm{max}}^{1}, &\displaystyle {\widehat{d}}_{{k}_{\mathrm{max}}^{1}}^{1}< { \left( \frac{\sum _{l=1}^{{k}_{\mathrm{max}}}\sqrt{{\widehat{d}}_{il}^{1}}}{{k}_{\mathrm{max}}^{1}-1-\sigma } \right) }^{2},\\ \displaystyle {l}^{{^{\prime}}}, &\displaystyle {\widehat{d}}_{i{l}^{{^{\prime}}}}^{1}< { \left( \frac{\sum _{l=1}^{{k}_{\mathrm{max}}}\sqrt{{\widehat{d}}_{il}^{1}}}{{k}_{\mathrm{max}}^{1}-1-\sigma } \right) }^{2}\mathrm{and}{\widehat{d}}_{i({l}^{{^{\prime}}}+1)}^{1}< { \left( \frac{\sum _{l=1}^{{k}_{\mathrm{max}}}\sqrt{{\widehat{d}}_{il}^{1}}}{{k}_{\mathrm{max}}^{1}-1-\sigma } \right) }^{2}, \end{array} \right. \end{eqnarray*}

where ${l}^{{^{\prime}}}< {k}_{\mathrm{max}}^{1}$. Based on Eq. (3), we obtain the neighbor set ${N}_{i}^{1}$ for spot *i*. The adjacency matrix ${A}^{1}=({A}_{i{i}^{{^{\prime}}}}^{1})_{s\times s}$ of the adaptive neighborhood graph based on gene expression is defined as follows

(4) \begin{eqnarray*}{A}_{i{i}^{{^{\prime}}}}^{1}= \left\{ \begin{array}{@{}ll@{}} \displaystyle \frac{\sum _{l=1}^{{k}_{max}}\sqrt{{\widehat{d}}_{il}^{1}}-({k}_{max}^{1}-1-\sigma )\sqrt{{d}_{i{i}^{{^{\prime}}}}^{1}}}{\sum _{l=1}^{{k}_{max}^{1}}{\widehat{d}}_{il}^{1}} , &\displaystyle \mathrm{if}{i}^{{^{\prime}}}\in {N}_{i}^{1}\mathrm{and}i\in {N}_{{i}^{{^{\prime}}}}^{1},\\ \displaystyle 0, &\displaystyle \mathrm{otherwise}. \end{array} \right. \end{eqnarray*}

The value of *σ* is selected *via* grid search. Specifically, *σ* is set to different values (0.1, 0.2, …, 0.9), and Louvain clustering is performed based on the resulting *A*^1^. The optimal *σ* is chosen as the one just before a sharp increase in the number of identified communities.

### Joint graph-regularized nonnegative matrix factorization model

Let *Y* = [*Y*_1_, *Y*_2_, …, *Y*_*i*_, …, *Y*_*s*_] ∈ ℝ^2×*s*^ denote the spatial coordinate matrix corresponding to the spatial transcriptomics data *X*, where *Y*_*i*_ = [*Y*_*i*1_, *Y*_*i*2_]^*T*^ represents the 2D coordinate vector of spot *i*. The spatial distance between spots *i* and *i*′ is defined as ${d}_{i{i}^{{^{\prime}}}}^{2}$, given by ${d}_{i{i}^{{^{\prime}}}}^{2}=\parallel {Y}_{i}-{Y}_{{i}^{{^{\prime}}}}\parallel =\sqrt{({Y}_{i1}-{Y}_{{i}^{{^{\prime}}}1})^{2}+({Y}_{i2}-{Y}_{{i}^{{^{\prime}}}2})^{2}}.$ The set of *k*^2^ nearest neighbors of spot *i* based on spatial location, denoted ${N}_{i}^{2}$, can be obtained. Define ${A}^{2}=({A}_{i{i}^{{^{\prime}}}}^{2})_{s\times s}$ as the adjacency matrix for spatial positions, where

(5) \begin{eqnarray*}{A}_{i{i}^{{^{\prime}}}}^{2}= \left\{ \begin{array}{@{}ll@{}} \displaystyle 1, &\displaystyle \mathrm{if}{i}^{{^{\prime}}}\in {N}_{i}^{2}\mathrm{and}i\in {N}_{{i}^{{^{\prime}}}}^{2},\\ \displaystyle 0, &\displaystyle \mathrm{otherwise}. \end{array} \right. \end{eqnarray*}

Let ${L}^{1}=({L}_{i{i}^{{^{\prime}}}}^{1})_{s\times s}$ denote the graph Laplacian matrix that reflects the topological relationships between gene expression neighbors. Specifically,

(6) \begin{eqnarray*}{L}^{1}={D}^{1}-{A}^{1},\end{eqnarray*}

(7) \begin{eqnarray*}{D}_{i{i}^{{^{\prime}}}}^{1}= \left\{ \begin{array}{@{}ll@{}} \displaystyle \sum _{{i}^{{^{\prime}}}}{A}_{i{i}^{{^{\prime}}}}^{1}, &\displaystyle i={i}^{{^{\prime}}},\\ \displaystyle 0, &\displaystyle \mathrm{otherwise.} \end{array} \right. \end{eqnarray*}

Similarly, let ${L}^{2}=({L}_{i{i}^{{^{\prime}}}}^{2})_{s\times s}$ be the Laplacian matrix describing the topological relationships among spatial neighbors

(8) \begin{eqnarray*}{L}^{2}={D}^{2}-{A}^{2},\end{eqnarray*}

(9) \begin{eqnarray*}{D}_{i{i}^{{^{\prime}}}}^{2}= \left\{ \begin{array}{@{}ll@{}} \displaystyle \sum _{{i}^{{^{\prime}}}}{A}_{i{i}^{{^{\prime}}}}^{2}, &\displaystyle i={i}^{{^{\prime}}},\\ \displaystyle 0, &\displaystyle \mathrm{otherwise.} \end{array} \right. \end{eqnarray*}

To obtain a low-dimensional representation of the spatial transcriptomics data, we first process the gene expression matrix *X*′ by selecting $\widetilde {m}$ highly variable genes (default $\widetilde {m}=2000$), and then normalize the matrix of selected genes to obtain $\widetilde {X}\in {\mathbb{R}}^{\widetilde {m}\times s}$. Based on this, we propose the Joint Graph-Regularized Nonnegative Matrix Factorization (JGR-NMF) model that integrates gene expression and spatial information

(10) \begin{eqnarray*}\min _{W\geq 0,H\geq 0}{\mathop{\parallel \widetilde {X}-WH\parallel }\nolimits }_{F}^{2}+{\alpha }_{1}\mathrm{Tr}(H{L}^{1}{H}^{T})+{\alpha }_{2}\mathrm{Tr}(H{L}^{2}{H}^{T}),\end{eqnarray*}

where ${\mathop{\parallel \cdot \parallel }\nolimits }_{F}^{2}$ denotes the Frobenius norm, and *α*_1_ and *α*_2_ are positive regularization parameters. $W\in {\mathbb{R}}^{\widetilde {m}\times f}$ and *H* ∈ ℝ^*f*×*s*^ are the basis matrix and coefficient matrix, respectively.

This model incorporates two adjacency matrices—*A*^1^ (adaptive adjacency matrix based on gene expression) and *A*^2^ (adjacency matrix based on spatial coordinates)—to simultaneously capture expression similarity and spatial proximity, thereby providing a more comprehensive representation of latent structures and spatial heterogeneity in spatial transcriptomics data. Notably, the matrix *H* represents the low-dimensional embedding of the spatial transcriptomics data. Therefore, clustering can be performed by applying the K-means algorithm to *H*.

### Model optimization

Following previous studies (Qiu et al., 2024; Wang, Bensmail & Gao, 2013), we adopt an alternating update algorithm to solve the optimization problem Eq. (10). First, we construct the following Lagrangian function

(11) \begin{eqnarray*}\begin{array}{@{}ll@{}} \displaystyle \mathcal{L}&\displaystyle ={\mathop{\parallel \widetilde {X}-WH\parallel }\nolimits }_{F}^{2}+{\alpha }_{1}\mathrm{Tr}(H{L}^{1}{H}^{T})+{\alpha }_{2}\mathrm{Tr}(H{L}^{2}{H}^{T})+\mathrm{Tr}(\Psi {W}^{T})+\mathrm{Tr}(\Phi {H}^{T})\\ \displaystyle &\displaystyle =\mathrm{Tr}(\widetilde {X}{\widetilde {X}}^{T})-2\mathrm{Tr}({H}^{T}{W}^{T}\widetilde {X})+\mathrm{Tr}(WH{H}^{T}{W}^{T})+{\alpha }_{1}\mathrm{Tr}(H{L}^{1}{H}^{T})\\ \displaystyle &\displaystyle  +{\alpha }_{2}\mathrm{Tr}(H{L}^{2}{H}^{T})+\mathrm{Tr}(\Psi {W}^{T})+\mathrm{Tr}(\Phi {H}^{T}), \end{array}\end{eqnarray*}

where Ψ and Φ are the Lagrange multipliers associated with the constraints *W* ≥ 0 and *H* ≥ 0, respectively. Taking the partial derivatives of the Lagrangian function $\mathcal{L}$ with respect to *W* and *H*, we obtain

(12) \begin{eqnarray*} \frac{\partial \mathcal{L}}{\partial W} =-2\widetilde {X}{H}^{T}+2WH{H}^{T}+\Psi ,\end{eqnarray*}

(13) \begin{eqnarray*} \frac{\partial \mathcal{L}}{\partial H} =-2{W}^{T}\widetilde {X}+2{W}^{T}WH+2H({\alpha }_{1}{L}^{1}+{\alpha }_{2}{L}^{2})+\Phi .\end{eqnarray*}

Setting Eqs. (12) and (13) to zero and multiplying both sides by *W*^*T*^ and *H*^*T*^, respectively, yield

(14) \begin{eqnarray*}-2\widetilde {X}{H}^{T}{W}^{T}+2WH{H}^{T}{W}^{T}+\Psi {W}^{T}=0,\end{eqnarray*}

(15) \begin{eqnarray*}-2{W}^{T}\widetilde {X}{H}^{T}+2{W}^{T}WH{H}^{T}+2H({\alpha }_{1}{L}^{1}+{\alpha }_{2}{L}^{2}){H}^{T}+\Phi {H}^{T}=0.\end{eqnarray*}

Substituting Eqs. (6) and (8) into (15) gives

(16) \begin{eqnarray*}-2({W}^{T}\widetilde {X}+{\alpha }_{1}H{A}^{1}+{\alpha }_{2}H{A}^{2}){H}^{T}+2({W}^{T}WH+{\alpha }_{1}H{D}^{1}+{\alpha }_{2}H{D}^{2}){H}^{T}+\Phi {H}^{T}=0.\end{eqnarray*}

According to the KKT conditions (Chen, Zeng & Pan, 2022), we have Ψ_*pq*_*W*_*pq*_ = 0 and Φ_*pq*_*H*_*pq*_ = 0. Therefore, Eqs. (14) and (16) can be rewritten as

(17) \begin{eqnarray*}(-\widetilde {X}{H}^{T}+WH{H}^{T})_{pq}{W}_{pq}=0,\end{eqnarray*}

(18) \begin{eqnarray*}(-{W}^{T}\widetilde {X}-{\alpha }_{1}H{A}^{1}-{\alpha }_{2}H{A}^{2}+{W}^{T}WH+{\alpha }_{1}H{D}^{1}+{\alpha }_{2}H{D}^{2})_{pq}{H}_{pq}=0.\end{eqnarray*}

From Eqs. (17) and (18), the update rules for the basis matrix *W* and the coefficient matrix *H* can be derived as

(19) \begin{eqnarray*}{W}_{pq}\leftarrow {W}_{pq} \frac{(\widetilde {X}{H}^{T})_{pq}}{(WH{H}^{T})_{pq}} ,\end{eqnarray*}

(20) \begin{eqnarray*}{H}_{pq}\leftarrow {H}_{pq} \frac{({W}^{T}\widetilde {X}+{\alpha }_{1}H{A}^{1}+{\alpha }_{2}H{A}^{2})_{pq}}{({W}^{T}WH+{\alpha }_{1}H{D}^{1}+{\alpha }_{2}H{D}^{2})_{pq}} .\end{eqnarray*}

As in previous works (Cai et al., 2011; Long et al., 2013), the update rules for dual-graph regularized non-negative matrix factorization are guaranteed to converge. Algorithm 1  provides the pseudocode for solving the proposed JGR-NMF.

 
_______________________ 
Algorithm 1 Joint Graph-Regularized Non-negative Matrix Factorization_____ 
Input: Gene expression count matrix X ∈ Rm×s; 
         Spatial location matrix Y ∈ R2×s; 
         Model parameters α1, α2; 
         Integers k1max, k2, m′, f; 
         Convergence threshold ϵ; 
         Maximum number of iterations MNI; 
Output: W and H 
 1:  Perform preliminary gene filtering and data normalization on X to obtain 
     the filtered matrix X′ ∈ Rm′×s 
      ; 
  2:  Select the top ^ m highly variable genes from X′ to obtain the reduced matrix 
      ^ X ∈ R^m×s; 
  3:  Construct adjacency matrices A1 and A2 using equations (4) and (5); 
  4:  Construct matrices D1 and D2 using equations (7) and (9); 
  5:  Construct Laplacian matrices L1 and L2 using equations (6) and (8); 
  6:  Randomly initialize W = W0 ∈ Rm′×f 
       ; 
  7:  Randomly initialize H = H0 ∈ Rf×s; 
  8:  for t = 1,2,...,MNI do 
 9:     Update W using equation (19); 
10:     Update H using equation (20); 
11:     Compute the error errH = ∥Ht − Ht−1∥∞; 
12:     if errH < ϵ then 
13:         Terminate iteration; 
14:         return  W and H; 
15:     end if 
16:  end for 
17:  Return: W and H______________________________________________________________    

### Computational complexity analysis

To evaluate the efficiency of JGR-NMF in processing large-scale data, we conducted a computational complexity analysis. The cost of the algorithm mainly comes from the alternating iterative updates of the base matrix *W* and the coefficient matrix *H*. Specifically, according to the update rule (19) (Equation (19)), the computational complexity of updating *W* is *O*(*m* × *s* × *f*). Similarly, according to the update rule (20) (Equation (20)), the complexity of updating *H* is also *O*(*m* × *s* × *f*). Theoretically, constructing the Laplacian matrices *L*^1^ and *L*^2^ has a complexity of *O*(*s*^2^). However, since the constructed adjacency matrices *A*^1^ and *A*^2^ are highly sparse (each spot has an average of only *k* neighbors and *k* ≪ *s*), they can be optimized to *O*(*s* × *k*) through sparse matrix operation techniques. Therefore, the overall complexity of a single iteration can be controlled at *O*(*m* × *s* × *f*). Assuming the number of iterations required for algorithm convergence is t, the total computational complexity of JGR-NMF is *O*(*t* × *m* × *s* × *f*).

## Results

### Comparison methods and evaluation metrics

To evaluate the performance of JGR-NMF, we benchmarked it against five existing methods: one non-spatial clustering approach (Seurat; Stuart et al., 2019) and four spatial clustering methods (SpaGCN; Hu et al., 2021), BANKSY (Singhal et al., 2024), MNMST (Wang, Liu & Ma, 2024), and spaMMCL (Liang et al., 2024b). For all methods, the number of clusters was set identically using default parameter configurations specified in their respective implementations. Performance was evaluated using three quantitative metrics: Adjusted Rand Index (ARI), Normalized Mutual Information (NMI), and Purity (PUR). The definitions of these metrics are as follows:

**(1) ARI.** ARI measures the consistency between the clustering results and the ground truth labels, adjusting for the chance grouping of elements. It ranges from −1 to 1, with values closer to 1 indicating higher clustering consistency. The ARI is defined as:

\begin{eqnarray*}ARI= \frac{RI-E(RI)}{\max \nolimits (RI)-E(RI)} , \end{eqnarray*}

where *RI* is the Rand Index, which reflects the similarity between the clustering results and true labels, *E*(*RI*) is the expected value of RI under random labeling, and max(*RI*) is the maximum value of RI. The Rand Index is computed as $RI= \frac{a+d}{a+b+c+d} ,$ where *a* is the number of sample pairs that are in the same cluster and have the same label, *b* is the number of pairs that are in the same label but in different clusters, *c* is the number of pairs that are in the same cluster but have different labels, and *d* is the number of pairs that are in different clusters and have different labels.

**(2) NMI.** NMI measures the amount of shared information between the predicted clusters and the ground truth labels, normalized to eliminate the influence of differing numbers of clusters and labels. It ranges from 0 to 1, with higher values indicating better clustering performance. Let there be *s* data points, and let *O* = {*O*_1_, *O*_2_, …, *O*_*K*_*t*__} denote the set of ground truth labels and $\widehat{O}=\{ {\widehat{O}}_{1},{\widehat{O}}_{2},\ldots ,{\widehat{O}}_{{K}_{f}}\} $ denote the set of predicted clusters, where *K*_*t*_ is the number of true classes and *K*_*f*_ is the number of predicted clusters. Then NMI is defined as

\begin{eqnarray*}NMI(\widehat{O},O)= \frac{2I(\widehat{O},O)}{H(\widehat{O})+H(O)} , \end{eqnarray*}

where $I(\widehat{O},O)={\mathop{\sum }\nolimits }_{u=1}^{{K}_{t}}{\mathop{\sum }\nolimits }_{v=1}^{{K}_{f}}P(u,v)\log \frac{P(u,v)}{P(u)P(v)} $ is the mutual information between $\widehat{O}$ and *O*, with *P*(*u*, *v*) denoting the joint probability that a sample belongs to both class *O*_*u*_ and cluster ${\widehat{O}}_{v}$, and *P*(*u*), *P*(*v*) denoting the marginal probabilities of *O*_*u*_ and ${\widehat{O}}_{v}$, respectively. The entropies *H*(*O*) and $H(\widehat{O})$ are given by $H(O)=-{\mathop{\sum }\nolimits }_{u=1}^{{K}_{t}}P({O}_{u})\log P({O}_{u})$ and $H(\widehat{O})=-{\mathop{\sum }\nolimits }_{v=1}^{{K}_{f}}P({\widehat{O}}_{v})\log P({\widehat{O}}_{v})$, respectively.

**(3) PUR.** Purity measures the extent to which each cluster contains samples from a single ground truth class. It ranges from 0 to 1, with higher values indicating more homogeneous clusters. It is defined as:

\begin{eqnarray*}PUR= \frac{1}{s} \sum _{v=1}^{{K}_{f}}\max _{u}{|}{\widehat{O}}_{v}\cap {O}_{u}{|}, \end{eqnarray*}

where *s* is the total number of samples, *K*_*f*_ is the number of predicted clusters, ${\widehat{O}}_{v}$ is the set of samples in the *v*-th predicted cluster, and *O*_*u*_ is the set of samples with the *u*-th true label. The term ${|}{\widehat{O}}_{v}\cap {O}_{u}{|}$ represents the number of samples in cluster ${\widehat{O}}_{v}$ that also belong to true class *O*_*u*_, and ${\max }_{u}{|}{\widehat{O}}_{v}\cap {O}_{u}{|}$ gives the number of samples in the majority class within cluster *v*.

### Spatial domain identification on the Breast Cancer-1 dataset

**Figure 3:**
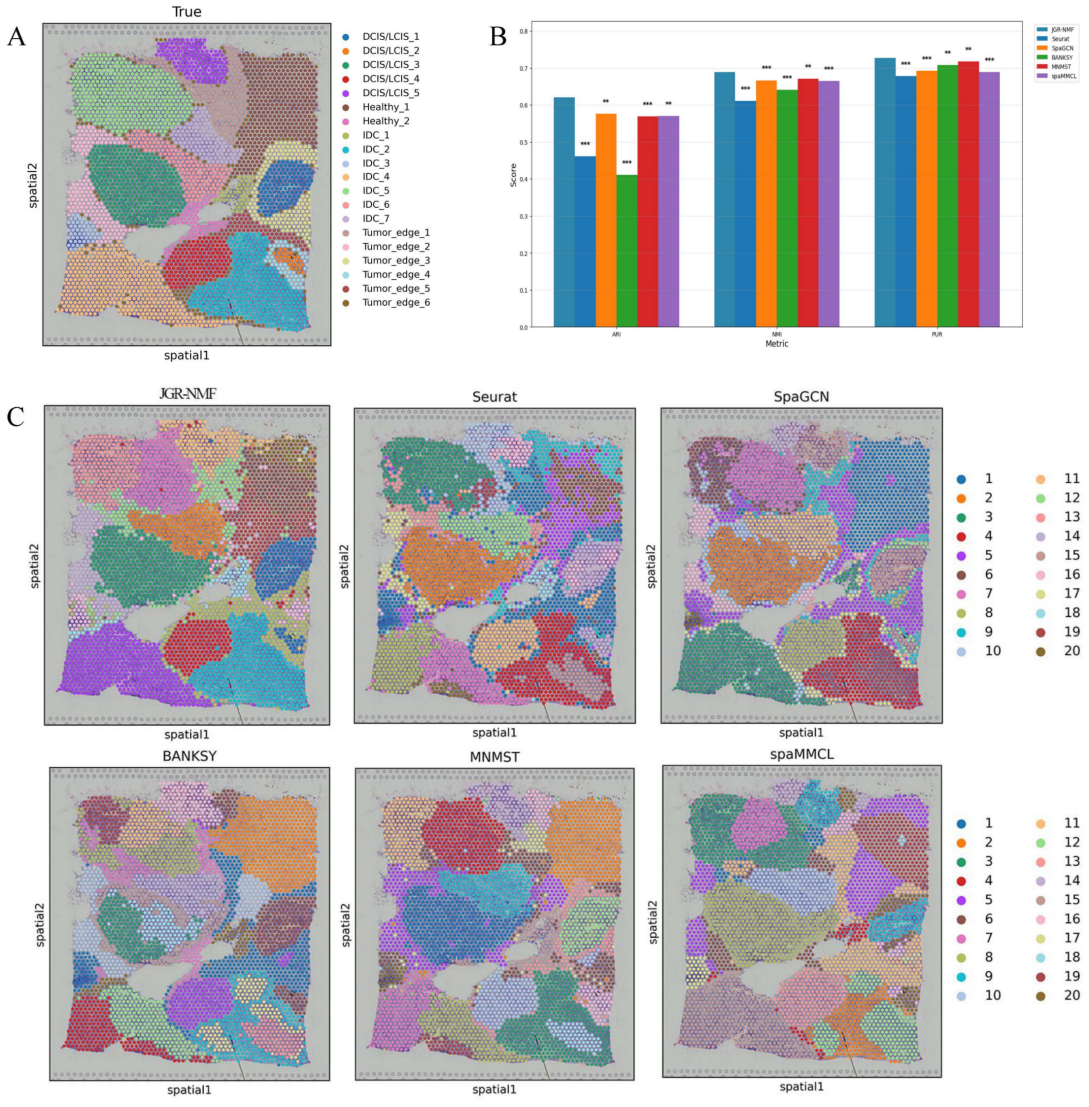
Spatial domain identification results on the Breast Cancer-1 dataset. (A) Manually annotated ground truth labels. (B) Bar chart comparing ARI, NMI, and PUR of different methods on the Breast Cancer-1 dataset. (C) Spatial domain identification visualizations.

To evaluate the spatial domain identification performance of JGR-NMF, we first conducted comparative experiments on the Breast Cancer-1 dataset against Seurat, SpaGCN, BANKSY, MNMST, and spaMMCL. Figure 3A shows the spatial distribution of 20 manually annotated ground truth labels in the Breast Cancer-1 dataset. Figure 3B shows the ARI, NMI and PUR scores of different methods on the Breast Cancer-1 dataset in a more intuitive bar chart format. The *x*-axis represents different evaluation metrics, the *y*-axis represents the scores, and different colors correspond to different methods. Higher values indicate better clustering performance.

The experimental results demonstrate that JGR-NMF achieved the highest ARI, NMI, and PUR scores on the Breast Cancer-1 dataset, indicating superior performance in spatial domain identification. Specifically, JGR-NMF achieved an ARI of 0.6210, which is 0.1599, 0.0452, 0.2097, 0.0523, and 0.0511 higher than those of Seurat, SpaGCN, BANKSY, MNMST, and spaMMCL, respectively. Its NMI value reached 0.6889, surpassing the compared methods by 0.0780, 0.0221, 0.0483, 0.0174, and 0.0235. Additionally, JGR-NMF achieved a PUR value of 0.7270, exceeding Seurat, SpaGCN, BANKSY, MNMST, and spaMMCL by 0.0487, 0.0342, 0.0187, 0.0094, and 0.0382, respectively. On the Breast Cancer-1 dataset, the hyperparameters *α*_1_, *α*_2_, ${k}_{max}^{1}$ and *σ* in JGR-NMF are 0.8, 0.1, 100 and 0.3 respectively. To evaluate the statistical significance of the performance difference of different methods on the Breast Cancer-1 dataset, we performed paired t-tests to quantify the difference between JGR-NMF and each baseline method in ARI, NMI and PUR indicators (Fig. 3B). The significance levels are marked as ∗ (*p* < 0.05), ∗∗ (*p* < 0.01), and ∗∗∗ (*p* < 0.001). The statistical test results indicate that JGR-NMF exhibits significant advantages over most baseline methods in terms of ARI, NMI, and PUR indicators.

Figure 3C further visualizes the spatial domain identification results of all six methods. These visualizations reveal the capacity of each method to recover the true spatial structure. JGR-NMF demonstrates a high degree of consistency with the ground truth labels in multiple regions, especially in “IDC_2”, “IDC_4”, “DCIS/LCIS 1”, “DCIS/LCIS 4”, and “Tumor_edge_1”, accurately capturing the spatial characteristics of these regions. In contrast, although other methods show some effectiveness in certain areas, their overall performance is inferior. For example, BANKSY performs relatively well in the “Healthy_1” region but suffers from fragmented clustering in other areas and fails to capture the overall spatial distribution of the true labels. Similarly, while SpaGCN and MNMST perform moderately well in some regions, they still fall short in complex labeling areas compared to JGR-NMF.

**Figure 4:**
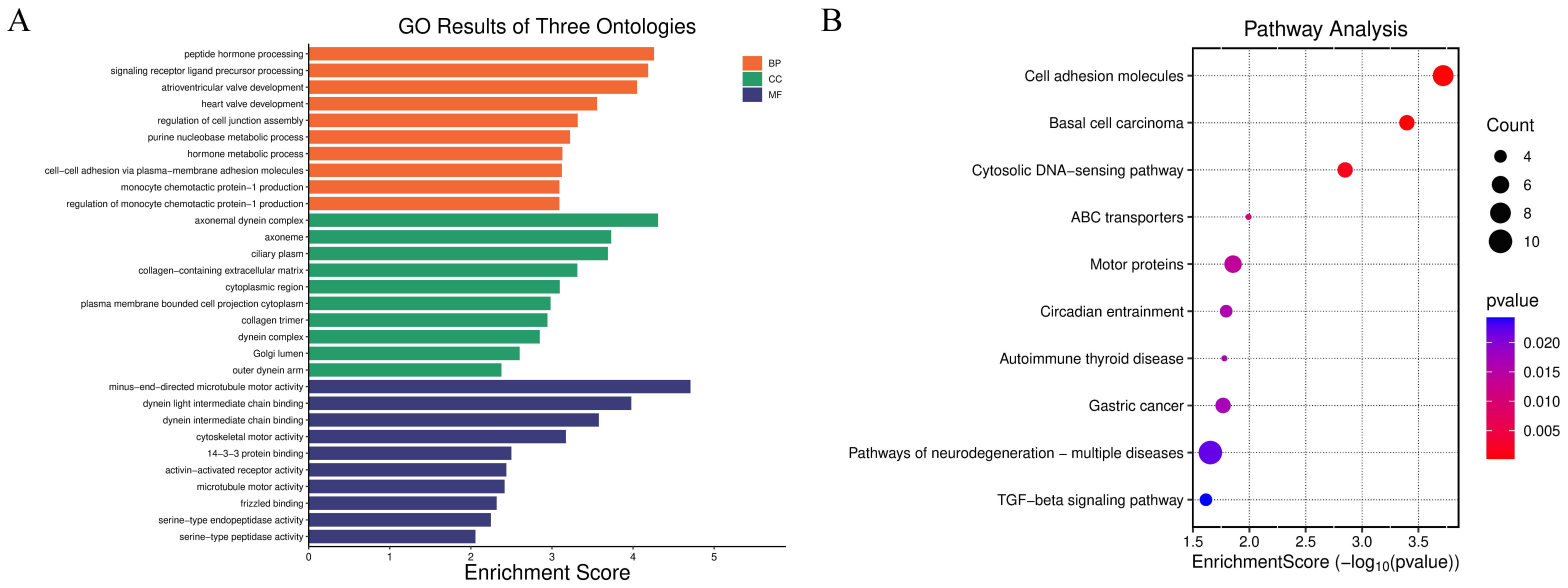
The results of gene enrichment analysis on the Breast Cancer-1 dataset. (A) Enrichment analysis results of BP, CC, and MF. (B) Bubble plot of KEGG pathway analysis. The larger the bubble, the more genes are involved, and the redder the color, the more significant the enrichment.

To further verify the biological rationality of the spatial domain identified by JGR-NMF on the Breast Cancer-1 dataset, we conducted differential expression analysis on the predicted spatial domain. Specifically, we adopted the non-parametric Wilcoxon rank-sum test to screen the significantly highly expressed genes that were expressed in at least 25% of the spots in each spatial domain and whose expression levels was |*log*2*FoldChange*| ≥ 0.25 as candidate marker genes. By comparing the correspondence between predicted clusters and real spatial domain labels, it was found that predicted cluster 5 is highly consistent in spatial distribution with the real histological label “IDC4” domain. Based on this, we screened the top 20 marker genes with the highest |*log*2*FoldChange*| values from cluster 5, and performed Gene Ontology (GO) enrichment analysis and Kyoto Encyclopedia of Genes and Genomes (KEGG) pathway enrichment analysis. The GO analysis results in Fig. 4A show that these genes are significantly enriched in multiple biological processes closely related to tumor progression, including “peptide hormone processing”, “cell–cell adhesion *via* plasma-membrane adhesion molecules”, and “heart valve development”. Cellular component terms were primarily associated with extracellular matrix structures and motor protein complexes, such as “collagen-containing extracellular matrix” and “dynein complex”. Molecular functions included “minus-end-directed microtubule motor activity” and “serine-type endopeptidase activity”. The KEGG pathway analysis in Fig. 4B further shows that these genes are significantly enriched in “Cell adhesion molecules”, “TGF-beta signaling pathway”, “Pathways of neurodegeneration-multiple diseases” and other pathways closely related to the occurrence and development of breast cancer. These results not only confirm the effectiveness of JGR-NMF in identifying biologically consistent spatial domains, but also reveal the potential molecular mechanisms of the IDC4 domain and its functional role in the tumor microenvironment.

### Spatial domain identification on the Breast Cancer-2 dataset

**Figure 5:**
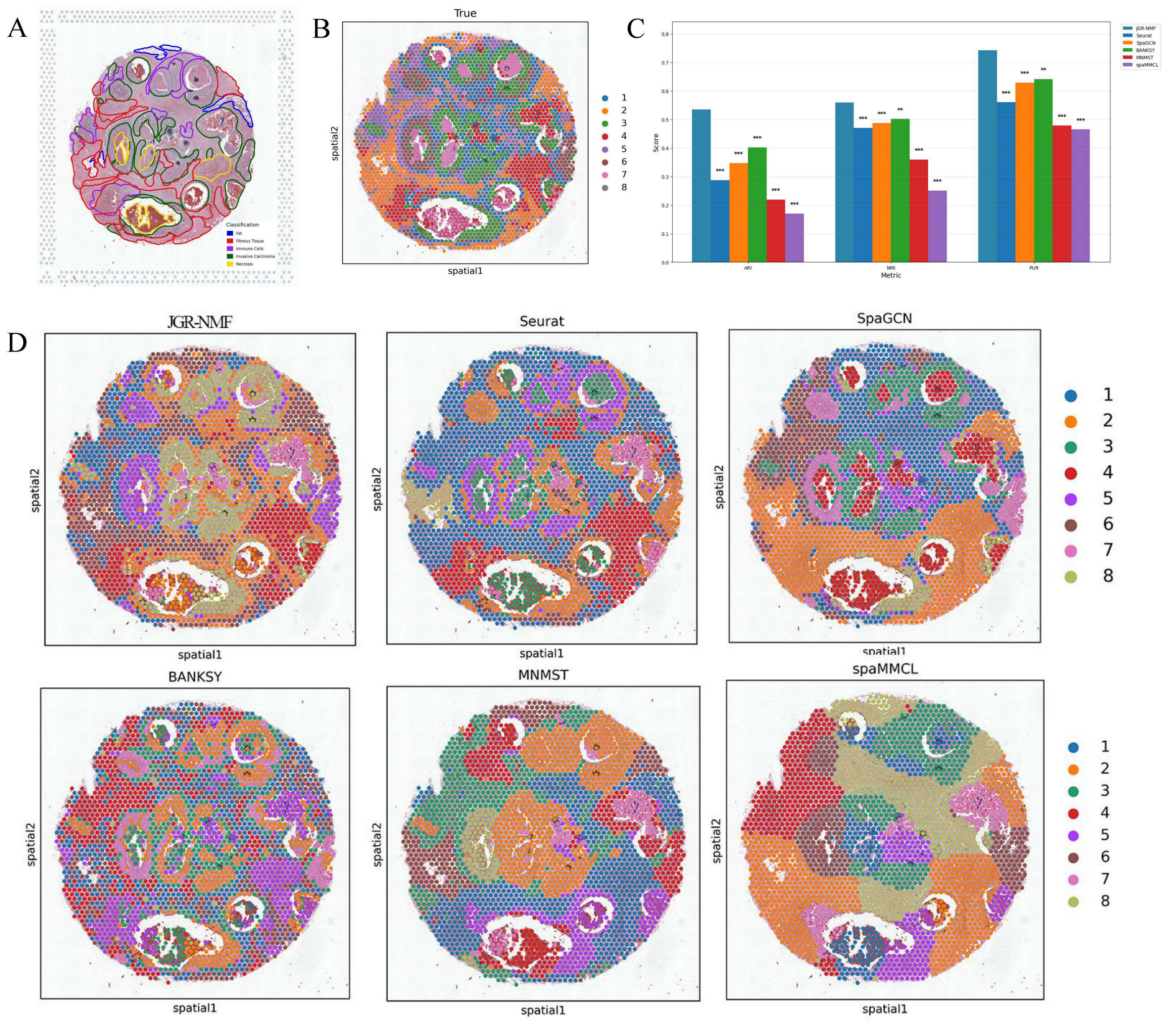
Spatial domain identification results of different methods on the Breast Cancer-2 dataset. (A) Manually annotated label structure of the Breast Cancer-2 dataset. (B) Ground truth label structure (GraphClust-based division). (C) Bar chart comparing ARI, NMI, and PUR of different methods on the Breast Cancer-2 dataset. (D) Visualization of spatial domain identification results by JGR-NMF, Seurat, SpaGCN, BANKSY, MNMST, and spaMMCL on the Breast Cancer-2 dataset.

To evaluate the performance of the JGR-NMF method in identifying spatial domains under different clustering numbers, we further conducted experiments on the Breast Cancer-2 dataset. Figures 5A and 5B show the manually annotated reference regions and the eight spatial domains divided by the GraphClust algorithm based on the 10x Visium platform, respectively. Since the manually annotated regions do not perfectly match the spot labels in the data, the eight regions identified by GraphClust are used as ground truth labels to evaluate the performance of different spatial domain identification methods.

Figure 5C visually displays the ARI, NMI, and PUR values of different methods on the Breast Cancer-2 dataset through a bar chart. The experimental results show that JGR-NMF performs best on the spatial domain identification task for the Breast Cancer-2 dataset, significantly outperforming other methods in terms of ARI, NMI, and PUR. Specifically, JGR-NMF achieves an ARI of 0.5354, which is higher than Seurat, SpaGCN, BANKSY, MNMST, and spaMMCL by 0.2479, 0.1876, 0.1334, 0.3163, and 0.3646, respectively. Its NMI value of 0.5599 exceeds those of Seurat, SpaGCN, BANKSY, MNMST, and spaMMCL by 0.0888, 0.0725, 0.0582, 0.2006, and 0.3087, respectively. Likewise, the PUR of JGR-NMF is 0.7431, surpassing Seurat, SpaGCN, BANKSY, MNMST, and spaMMCL by 0.1827, 0.1136, 0.1013, 0.2645, and 0.2780, respectively. For the Breast Cancer-2 dataset, we set the JGR-NMF hyperparameters to *α*_1_ = 0.2, *α*_2_ = 1, ${k}_{max}^{1}=50$, and *σ* = 0.5. In addition, the *t*-test results in Fig. 5C indicate that JGR-NMF is significantly superior to other comparative methods in terms of ARI, NMI, and PUR indicators.

The spatial domain identification visualizations of the six methods on the Breast Cancer-2 dataset are shown in Fig. 5D. From these visualizations, it is evident that the domains identified by JGR-NMF are more consistent with the ground truth labels, especially in fibrous tissue and invasive cancer regions corresponding to ground truth labels “2”, “3”, “4”, and “6” (Fig. 5A), where JGR-NMF exhibits strong identification ability for cancer-related areas.

In contrast, although some methods have partial overlap with the ground truth, their overall performance is suboptimal. For example, Seurat tends to produce overly concentrated clusters, with most spots assigned to a single cluster, leading to low discrimination. BANKSY, on the other hand, produces overly fragmented clusters and fails to form meaningful spatial structures. SpaGCN, MNMST, and spaMMCL tend to aggregate spatially adjacent regions into large blocks, resulting in boundaries that deviate from the ground truth.

In conclusion, JGR-NMF not only achieves the best performance in terms of quantitative evaluation metrics such as ARI, NMI, and PUR but also demonstrates superior spatial domain identification in visual inspection, outperforming Seurat, SpaGCN, BANKSY, MNMST, and spaMMCL.

**Figure 6:**
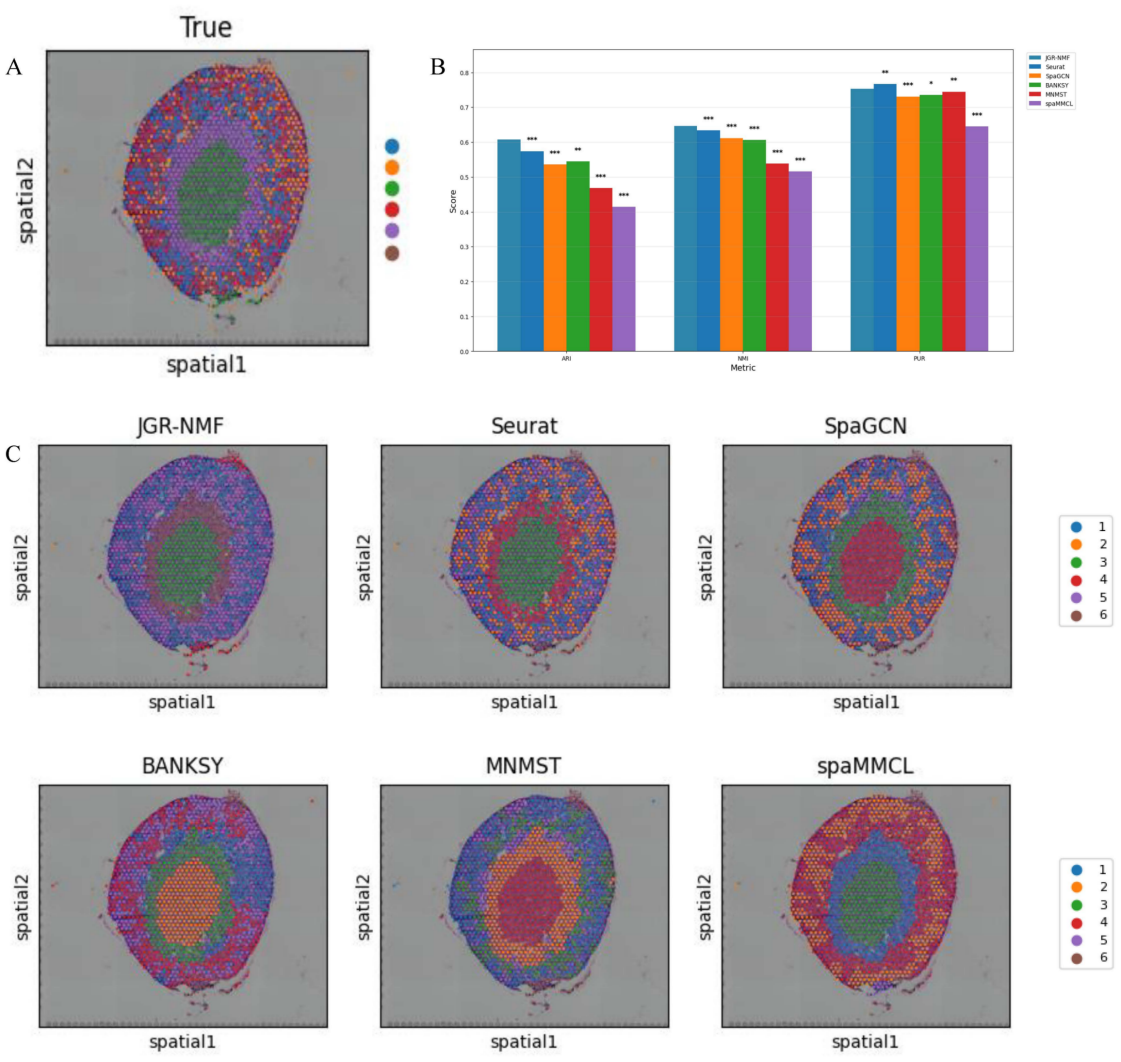
Spatial domain identification results on the Mouse Kidney Dataset. (A) Ground truth label structure of the Mouse Kidney dataset. (B) Bar chart showing the ARI, NMI and PUR scores of different methods on the Mouse Kidney dataset. (C) Spatial domain identification visualizations of JGR-NMF, Seurat, SpaGCN, BANKSY, MNMST, and spaMMCL on the Mouse Kidney dataset.

### Spatial domain identification on the mouse kidney dataset

To further evaluate the generalization performance of JGR-NMF method in identifying spatial domains on different datasets, we conducted comparative experiments with Seurat, SpaGCN, BANKSY, MNMST, and spaMMCL methods on the Mouse Kidney dataset. Figure 6A shows the spatial distribution of six ground truth labels manually annotated in the Mouse Kidney dataset. Similar to the visualization results on the breast cancer dataset, we used the bar chart in Fig. 6B to compare the ARI, NMI and PUR scores of different methods on the Mouse Kidney dataset. The experimental results indicate that JGR-NMF performs the best in the spatial domain identification task on the Mouse Kidney dataset. Specifically, the ARI value of JGR-NMF is 0.6074, which is 0.0333, 0.0718, 0.0624, 0.1416, and 0.1934 higher than Seurat, SpaGCN, BANKSY, MNMST, and spaMMCL, respectively. The NMI value of JGR-NMF is 0.6463, which is 0.0124, 0.0345, 0.0399, 0.1078, and 0.1308 higher than Seurat, SpaGCN, BANKSY, MNMST, and spaMMCL, respectively. Similarly, the PUR of JGR-NMF is 0.7531, which is 0.0222, 0.0174, 0.0083, and 0.1078 higher than SpaGCN, BANKSY, MNMST, and spaMMCL, respectively. Although Seurat has a slight advantage in purity indicators on the Mouse Kidney dataset, the JGR-NMF method is the best based on the comprehensive ARI and NMI indicators. On the Mouse Kidney dataset, the hyperparameters for JGR-NMF were set as follows: *α*_1_ = 0.001, *α*_2_ = 0.999, ${k}_{max}^{1}=45$, and *σ* = 0.6. In addition, the *t*-test results in Fig. 6B indicate that JGR-NMF is significantly superior to other comparative methods in ARI, NMI, and PUR indicators on the Mouse Kidney dataset.

The spatial domain identification results of all six methods are presented in Fig. 6C. It can be observed that JGR-NMF shows a high degree of consistency with the ground truth labels of multiple domains. In contrast, although other methods demonstrate some effectiveness in certain domains, their overall performance is poorer.

### Spatial domain identification on the mouse embryo dataset

To evaluate the clustering performance of JGR-NMF on datasets from different spatial transcriptomics platforms, we conducted comparative experiments on the seqFISH Mouse Embryo dataset against Seurat, SpaGCN, BANKSY, MNMST, and spaMMCL. The primary difference between seqFISH and 10x Visium data lies in the much higher single-cell resolution of seqFISH, which enables the direct detection of gene expression at the single-cell level while preserving spatial positional information. The high resolution of seqFISH data provides a suitable condition to verify the fine-grained performance of JGR-NMF.

**Figure 7:**
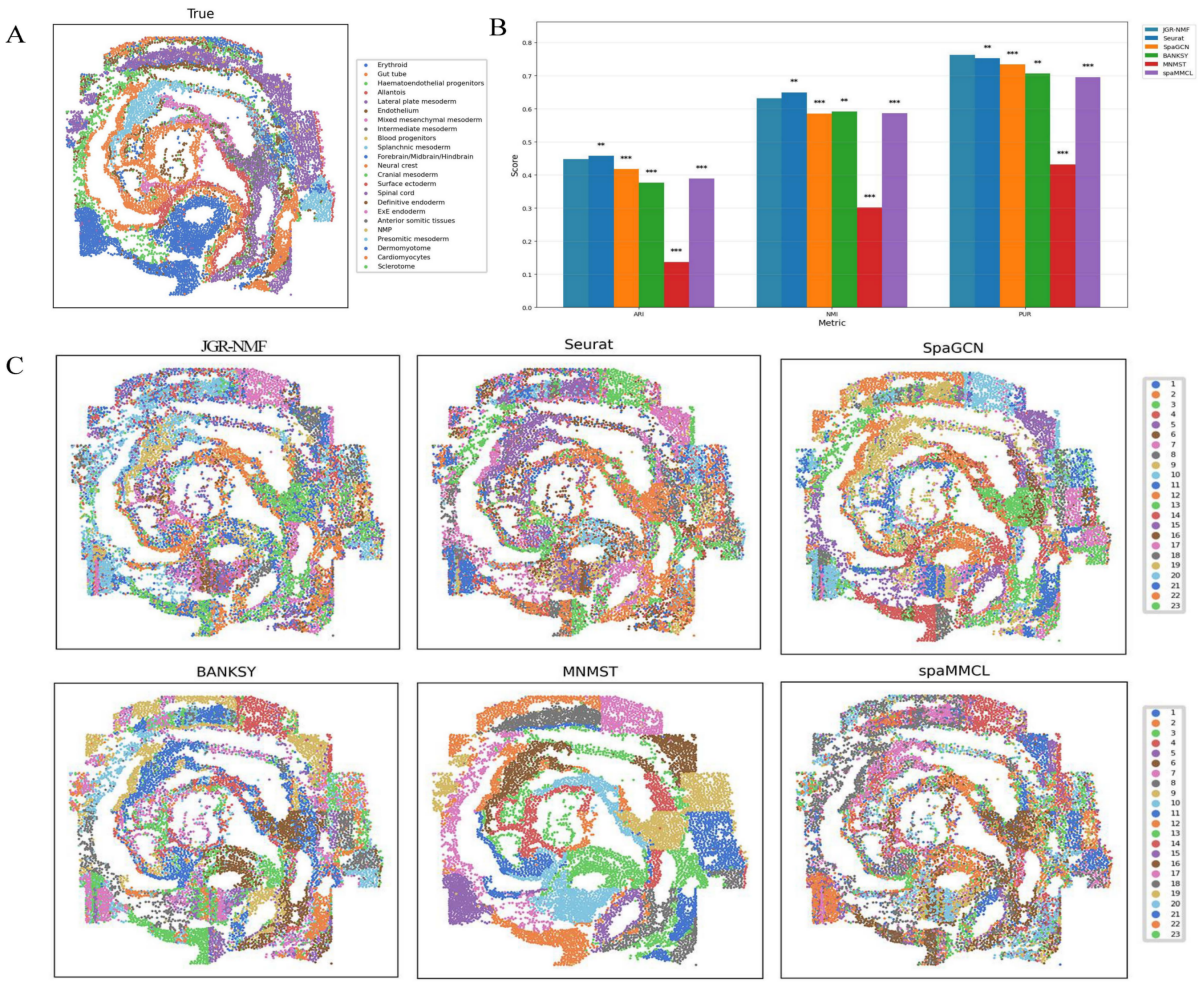
Spatial domain identification results of different methods on the Mouse Embryo dataset. (A) Ground truth label structure of the Mouse Embryo dataset. (B) Bar chart comparing ARI, NMI, and PUR results of different methods on the Mouse Embryo dataset. (C) Spatial domain identification visualizations of JGR-NMF, Seurat, SpaGCN, BANKSY, MNMST, and spaMMCL on the Mouse Embryo dataset.

Figure 7A shows the spatial distribution of 23 ground truth labels in the Mouse Embryo dataset. These regions exhibit curved, band-like, and relatively scattered spatial patterns. To better compare the performance of different methods, the ARI, NMI, and PUR scores of all methods are presented in Fig. 7B, where the horizontal axis represents different indicators and the vertical axis represents scores. Different methods are color-coded for clarity. The experimental results show that JGR-NMF achieves the highest PUR score on the Mouse Embryo dataset, and it also obtains the highest ARI and NMI values among all methods except for Seurat. Specifically, JGR-NMF achieves an ARI of 0.4478, which is higher than SpaGCN, BANKSY, MNMST, and spaMMCL by 0.0300, 0.0714, 0.3108, and 0.0589, respectively. In terms of NMI, JGR-NMF scores 0.6319, exceeding SpaGCN, BANKSY, MNMST, and spaMMCL by 0.0459, 0.0406, 0.3308, and 0.0457, respectively. Moreover, JGR-NMF achieves a PUR score of 0.7270, which is 0.0107, 0.0293, 0.0566, 0.3312, and 0.0674 higher than Seurat, SpaGCN, BANKSY, MNMST, and spaMMCL, respectively. On the Mouse Embryo dataset, we adopted the following hyperparameter configuration for JGR-NMF: *α*_1_ = 0.1, *α*_2_ = 0.6, ${k}_{max}^{1}=52$, and *σ* = 0.3. Paired *t*-test was adopted to evaluate the statistical significance of performance differences between different methods on a mouse embryo dataset. The statistical test results in Fig. 7B indicate that JGR-NMF exhibits significant advantages over most baseline methods in terms of ARI, NMI, and PUR indicators.

Furthermore, the spatial domain identification results of the six methods on the Mouse Embryo dataset are visualized in Fig. 7C, where different colors correspond to different labels. From the visual results, we observe that the spatial domains identified by JGR-NMF are more consistent with the ground truth labels and display clear band-like structures.

Although Seurat slightly outperforms JGR-NMF in ARI and NMI (by 0.0097 and 0.0179, respectively), this can be attributed to the fact that Seurat is a non-spatial clustering method and is inherently better suited for single-cell resolution data. Given that seqFISH data are essentially single-cell data, Seurat performs slightly better in this context.

However, it is worth noting that Fig. 7C shows that despite Seurat’s slight lead, its results do not effectively capture finer banded regions, whereas JGR-NMF performs better in this regard. Specifically, JGR-NMF clearly identifies multiple band-like regions that closely match the ground truth spatial layout. In contrast, MNMST shows significant clustering in red blood cell regions but produces blocky clusters in other areas, which deviate substantially from the ground truth. Additionally, SpaGCN, BANKSY, and spaMMCL exhibit weaker abilities in identifying band-like regions, with more scattered clustering results and poorer correspondence with the ground truth.

In conclusion, although Seurat shows a slight advantage in single-cell clustering tasks, JGR-NMF demonstrates stronger capabilities in identifying more complex spatial distribution patterns, especially in accurately recognizing spatial domains with complex banded structures.

The personal computer configuration used in the experiment is as follows: (1) CPU: Intel Core i5-8265U; (2) Memory: 16GB RAM; (3) Software: R (version 4.3.2; R Core Team, 2023) and Python (version 3.12). Tables 2 and 3 list the total runtime and memory usage of all methods on four datasets. On all datasets, the total running time of JGR-NMF is significantly shorter than that of deep learning based methods (SpaGCN and spaMMCL). Although the running time of JGR-NMF is slightly longer than Seurat, BANKSY, and SpaGCN, considering its significant advantage in clustering accuracy, this additional time cost is reasonable and acceptable in practical applications. In addition, JGR-NMF maintained a low memory usage in all experiments, especially on large-scale Mouse Embryo datasets. This was mainly due to the model’s approximately linear computational complexity and the optimized operations on sparse matrices.

**Table 2:** The total running time (min) of all methods on the four datasets.

	JGR-NMF	Seurat	SpaGCN	BANKSY	MNMST	spaMMCL
Breast cancer-1	48	12	120	25	40	140
Breast cancer-2	35	8	70	18	30	90
Mouse kidney	20	5	45	12	18	50
Mouse embryo	150	45	380	80	150	465

**Table 3:** The memory usage (MB) of all methods on the four datasets.

	JGR-NMF	Seurat	SpaGCN	BANKSY	MNMST	spaMMCL
Breast cancer-1	6,800	5,300	8,000	5,500	6,700	7,500
Breast cancer-2	5,300	3,800	6,500	3,800	5,200	6,200
Mouse kidney	3,000	2,200	4,200	2,800	3,400	4,500
Mouse embryo	15,000	12,000	18,000	13,000	1,400	17,000

### Ablation study

To comprehensively evaluate the impact of two regularization terms on the performance of JGR-NMF, we conducted detailed comparative experiments. These regularization terms were constructed based on gene expression and spatial location information, respectively. Experiments were performed on multiple datasets, including the Breast Cancer-1 dataset, Breast Cancer-2 dataset, and the Mouse Embryo dataset. Let and denote the two regularization terms constructed according to Equations (6) and (8), respectively. By combining these regularization terms, we assessed the influence of different topological structures on the clustering performance of JGR-NMF.

Tables 4, 5 and 6 present the ablation study results on the Breast Cancer-1, Breast Cancer-2, and Mouse Embryo datasets, respectively, including three key metrics: ARI, NMI, and PUR. These metrics effectively reflect the clustering accuracy and spatial domain identification performance of different model variants. The experimental results show that the model integrating both gene expression-based and spatial position-based neighbor topologies—constructed using adaptive neighborhood mechanisms—achieves the highest ARI, NMI, and PUR scores across all datasets, demonstrating the advantage of the proposed method in handling complex spatial transcriptomics data.

**Table 4:** Ablation results on the breast cancer-1 dataset.

	ARI	NMI	PUR
	0.5145	0.5840	0.6345
	0.6142	0.6814	0.7212
+	**0.6210**	**0.6889**	**0.7270**

**Notes.**

Bold values represent the optimal results for each evaluation metric.

**Table 5:** Ablation results on the breast cancer-2 dataset.

	ARI	NMI	PUR
	0.3419	0.4514	0.5790
	0.5297	0.5514	0.7419
+	**0.5354**	**0.5599**	**0.7431**

**Notes.**

Bold values represent the optimal results for each evaluation metric.

**Table 6:** Ablation results on the mouse embryo dataset.

	ARI	NMI	PUR
	0.4294	0.6201	0.7603
	0.4318	0.6166	0.7306
+	**0.4478**	**0.6319**	**0.7633**

**Notes.**

Bold values represent the optimal results for each evaluation metric.

On the Breast Cancer-1 dataset, the model with both regularization terms outperforms the model using only by 0.0068, 0.0076, and 0.0058 in ARI, NMI, and PUR, respectively, indicating that the integration of spatial and gene expression information significantly improves clustering results. This trend is further confirmed on the Breast Cancer-2 dataset, where ARI, NMI, and PUR increase by 0.0161, 0.0154, and 0.0327, respectively, showing that in more complex datasets, the integrated model can better exploit spatial structural features.

Moreover, on the Mouse Embryo dataset, the combined model also demonstrates superior performance, with ARI, NMI, and PUR increasing by 0.0056, 0.0085, and 0.0012, respectively, compared to using only . These results further verify the ability of model to stably improve clustering accuracy under different experimental settings. Overall, although the model constructed using only spatial information performs slightly better than using only gene expression, their combination significantly enhances the overall performance. This finding suggests that the joint use of spatial and gene expression information can effectively improve the applicability and accuracy of clustering methods on complex datasets, especially in tasks that require leveraging spatial distribution patterns, where the regularization-integrated model exhibits strong advantages.

## Conclusions

To improve the accuracy and robustness of spatial domain identification, this study proposed a novel method named JGR-NMF, which integrates gene expression and spatial location information by constructing an adaptive neighbor graph based on gene expression and a spatial adjacency matrix derived from spatial coordinates. This approach effectively captures both gene expression correlations and spatial relationships between spots, enabling more precise spatial domain identification. The model design incorporates graph regularization, which explicitly incorporates both gene expression similarity and spatial neighborhood structure into the optimization process. This integration significantly enhances the accuracy and robustness of spatial domain identification. We validated the effectiveness of JGR-NMF using two breast cancer datasets, one Mouse Kidney dataset and one Mouse Embryo dataset. Experimental results demonstrate that JGR-NMF exhibits superior performance in spatial domain identification tasks compared to several existing mainstream methods (Seurat, SpaGCN, BANKSY, MNMST, and spaMMCL). Specifically, JGR-NMF achieves finer and more accurate spatial domain identification, particularly in datasets characterized by complex tissue architecture. Ablation studies further evaluated the contribution of the graph regularization terms to the performance of JGR-NMF. Results indicate that removing the graph regularization leads to a significant decline in domain identification accuracy, confirming the critical role of these terms in enhancing spatial domain identification.

## Supplemental Information

10.7717/peerj.20585/supp-1 Supplemental Information 1 Data access instructions

## Additional Information and Declarations

### Data Availability

The following information was supplied regarding data availability: JGR-NMF is implemented in R. The source code and documentation are available at GitHub and Zenodo: - https://github.com/97-cute/JGR-NMF. - hjx. (2025). JGR-NMF: joint graph-regularized non-negative matrix factorization for spatial domain identification (V1.0.0). Zenodo. https://doi.org/10.5281/zenodo.17709368
The seqFISH Mouse Embryo dataset is available at: https://content.cruk.cam.ac.uk/jmlab/SpatialMouseAtlas2020. Please see the access instructions in the Supplemental Files to access the following datasets: The Breast Cancer-1 Dataset is available at: https://www.10xgenomics.com/datasets/human-breast-cancer-block-a-section-1-1-standard-1-1-0. The Breast Cancer-2 Dataset is available at: https://www.10xgenomics.com/datasets/human-breast-cancer-ductal-carcinoma-in-situ-invasive-carcinoma-ffpe-1-standard-1-3-0. The Mouse Kidney Dataset is available at: https://www.10xgenomics.com/datasets/mouse-kidney-section-coronal-1-standard-1-1-0.
