# Supplementary material for: JGR-NMF: joint graph-regularized non-negative matrix factorization for spatial domain identification
Source: PeerJ. 2026 Feb 10;14:e20585. doi: 10.7717/peerj.20585 (PMC12903897; doi:10.7717/peerj.20585)

1. Breast Cancer-1 dataset

Step(1): Copy the link (https://www.10xgenomics.com/datasets/human-breast-ca

ncer-block-a-section-1-1-standard-1-1-0) and open it in your browser. You will see the following page:


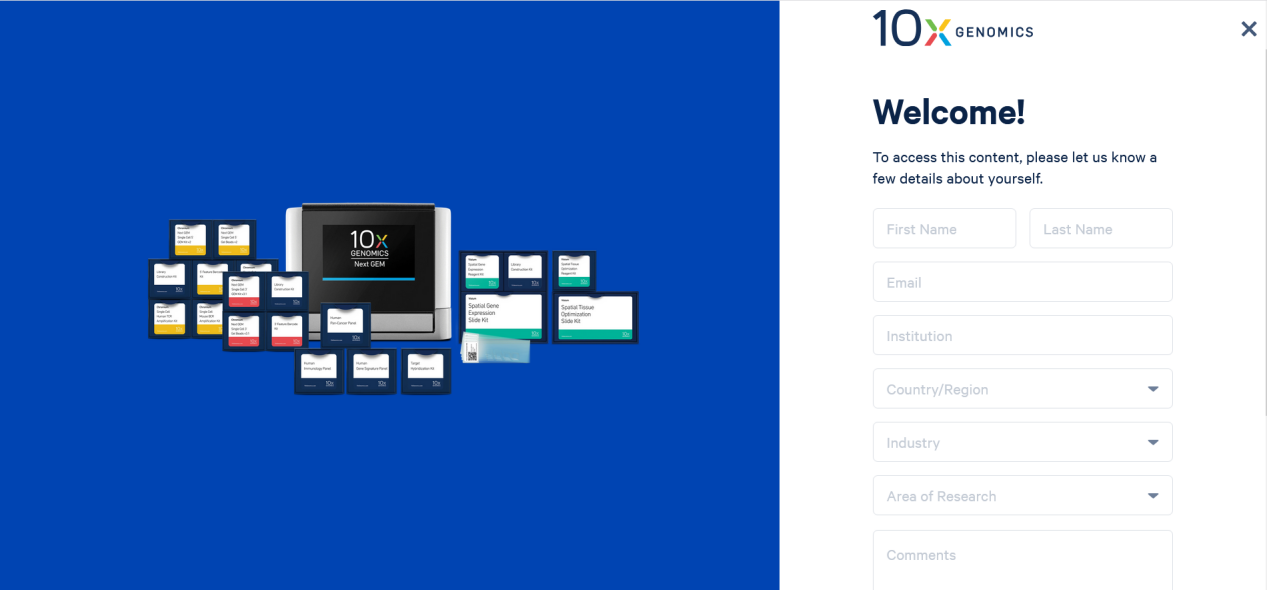


Step(2): Fill in the required personal information according to the prompts on the page opened in step (1). Here is an example of filling out:

**First Name**: Jiuxi

**Last Name**: Huang

**Email**: huangjiuxi@126.com

**Institution**: Henan Normal University

**Country/Region**: China/河南Henan

**Industry**: University

**Area of Research**: Genetic Disease

After filling out, please check the two option boxes at the bottom of the page. Then click the “Submit” button. The page will redirect to the following interface:


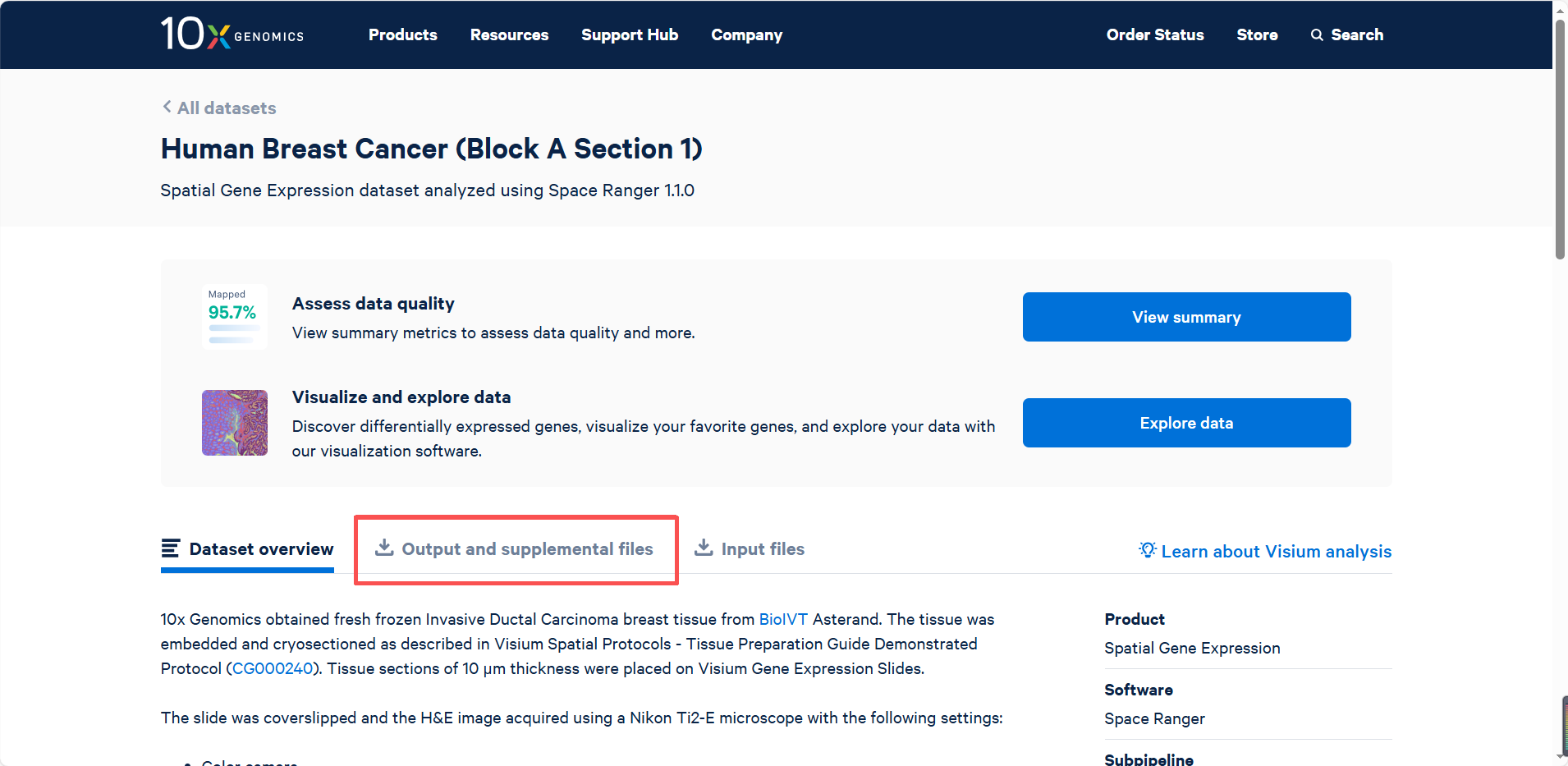


Step(3): On the page obtained in Step (2), click the “Output and supplemental files” option. You can then view and download the Breast Cancer-1 Dataset files, as shown in the following figure.


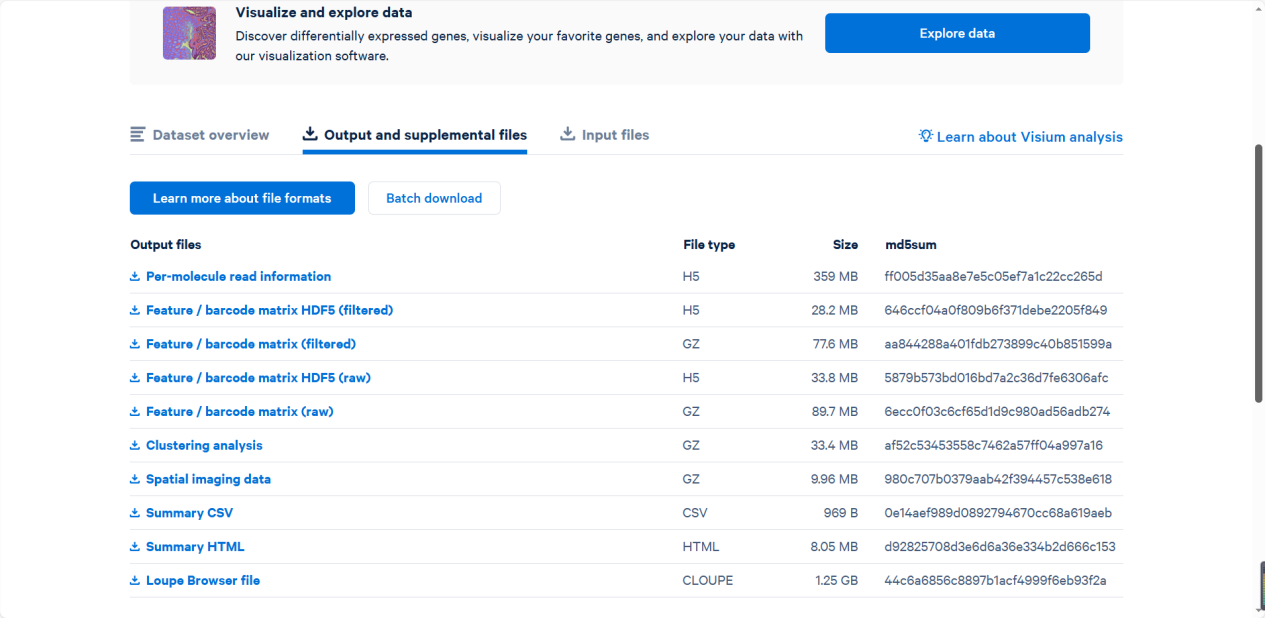


1. Breast Cancer-2 dataset

The process of obtaining the Breast Cancer-2 dataset is similar to that of Breast Cancer-1 dataset. First, copy and open the link ([https://www.10xgenomics.com/ datasets/human-breast-cancer-ductal-carcinoma-in-situ-invasive-carcinoma-ffpe-1-standard-1-3-0](https://www.10xgenomics.com/datasets/human-breast-cancer-ductal-carcinoma-in-situ-invasive-carcinoma-ffpe-1-standard-1-3-0)). Then, fill in your personal information on the page and submit the form. After submission, click the “Output and supplemental files” option to access the download interface for the Breast Cancer-2 dataset, as shown in the figure below.


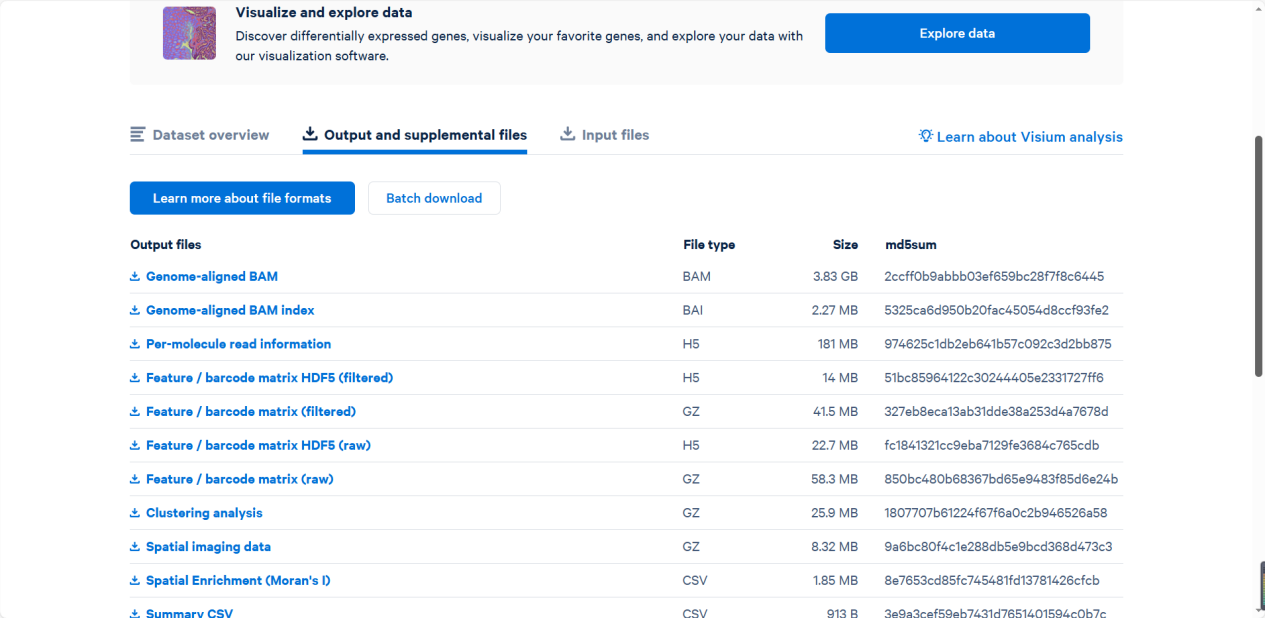


1. Mouse Kidney Dataset

The process of obtaining the Mouse Kidney Dataset is similar to that of Breast Cancer-1 dataset. First, copy and open the link (https://www.10xgenomics.com/ datasets/mouse-kidney-section-coronal-1-standard-1-1-0). Then, fill in your personal information on the page and submit the form. After submission, click the “Output and supplemental files” option to access the download interface for the Breast Cancer-2 dataset, as shown in the figure below.


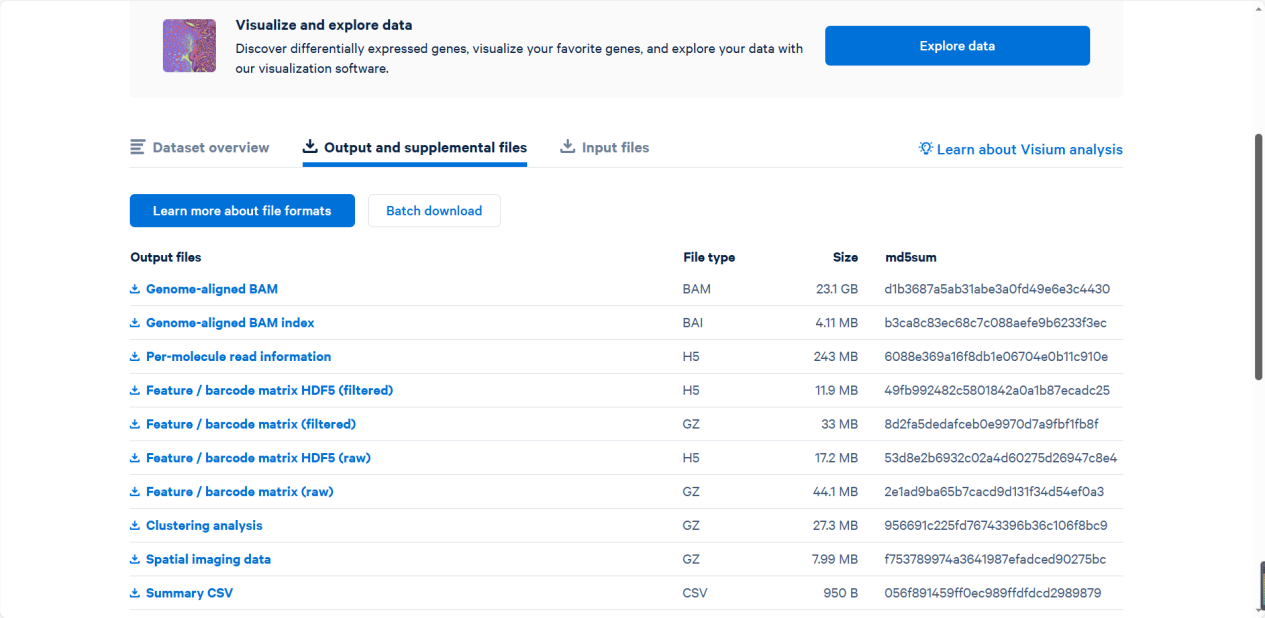

Supplement: Supplemental Information 1 [file peerj-14-20585-s001.docx]
